# “There is nothing so practical as a good theory”: a pragmatic guide for selecting theoretical approaches for implementation projects

**DOI:** 10.1186/s12913-018-3671-z

**Published:** 2018-11-14

**Authors:** Elizabeth A. Lynch, Alison Mudge, Sarah Knowles, Alison L. Kitson, Sarah C. Hunter, Gill Harvey

**Affiliations:** 1 Adelaide Nursing School, Faculty of Health and Medical Sciences, The University of Adelaide, Adelaide, South Australia 5000 Australia; 2 Internal Medicine and Aged Care, Royal Brisbane and Women’s Hospital, Corner Butterfield Street and Bowen Bridge Road, Herston, QLD 4019 Australia; 3 Alliance Manchester Business School – People, Management and Organisation Division, The University of Manchester, Oxford Road, Manchester, M13 9PL UK; 4 College of Nursing and Health Sciences, Flinders University, Sturt Road, Bedford Park, South Australia 5042 Australia

**Keywords:** Evidence-based practice, Implementation, Knowledge translation, Theory-informed

## Abstract

**Background:**

A multitude of theories, models and frameworks relating to implementing evidence-based practice in health care exist, which can be overwhelming for clinicians and clinical researchers new to the field of implementation science. Clinicians often bear responsibility for implementation, but may be unfamiliar with theoretical approaches designed to inform or understand implementation.

**Main text:**

In this article, a multidisciplinary group of clinicians and health service researchers present a pragmatic guide to help clinicians and clinical researchers understand what implementation theories, models and frameworks are; how a theoretical approach to implementation might be used; and some prompts to consider when selecting a theoretical approach for an implementation project. Ten commonly used and highly cited theoretical approaches are presented, none of which have been utilised to their full potential in the literature to date. Specifically, theoretical approaches tend to be applied retrospectively to evaluate or interpret findings from a completed implementation project, rather than being used to plan and design theory-informed implementation strategies which would intuitively have a greater likelihood of success. We emphasise that there is no right or wrong way of selecting a theoretical approach, but encourage clinicians to carefully consider the project’s purpose, scope and available data and resources to allow them to select an approach that is most likely to “value-add” to the implementation project.

**Conclusion:**

By assisting clinicians and clinical researchers to become confident in selecting and applying theoretical approaches to implementation, we anticipate an increase in theory-informed implementation projects. This then will contribute to more nuanced advice on how to address evidence-practice gaps and ultimately to contribute to better health outcomes.

**Electronic supplementary material:**

The online version of this article (10.1186/s12913-018-3671-z) contains supplementary material, which is available to authorized users.

## Background

Clinicians and clinical researchers usually have expert knowledge about evidence-based interventions for different clinical conditions. While some health professionals may have experience of implementing evidence-based interventions in their own practice or overseeing a change in practice by health professionals directly under their supervision, many are not familiar or confident with the current evidence regarding how to effectively, efficiently and sustainably implement evidence-based interventions into routine clinical practice.

Implementation science has been defined as “the scientific study of methods to promote the systematic uptake of research findings and other evidence-based practices into routine practice, and, hence, to improve the quality and effectiveness of health services” [1], and recognises that strong evidence alone is not sufficient to change practice. Researchers from a multitude of backgrounds have proposed different approaches to predicting, guiding and explaining how evidence is implemented, drawing on practical experience, observation, empirical study and the development and synthesis of an eclectic range of theories about individual, group and organisational change. This has resulted in a plethora of implementation frameworks, models, and theories; recent data suggest more than 100 theoretical approaches are being used by implementation researchers [2].

Use of established implementation theories, models or frameworks can help implementation researchers through contributing to new and more nuanced knowledge about how and why implementation succeeds or fails [3]. For clinicians, implementation theories, models and frameworks can be applied so new initiatives are planned, implemented and evaluated more systematically, which may enhance the success, sustainability and scalability of the project. When we consciously move our thinking from implicit assumptions about how we think implementation works to making our thinking more explicit and structured through the application of an established theoretical approach, then we might be able to be more objective and more creative about our approach to planning, guiding and evaluating.

Despite the growing recognition of the need to use theory to inform implementation programs [3], many clinicians and clinical researchers are unfamiliar with theories of implementation and behaviour change. For instance, in Australia in 2012, the majority of medical, nursing and allied health professionals who had successfully applied for National Health and Medical Research Council (NHMRC) Translating Research Into Practice (TRIP) fellowships had no previous experience using implementation theories or frameworks [4].

While previous manuscripts about implementation theories, models and frameworks are helpful for researchers with good background knowledge in implementation science to make sense of the different theoretical approaches available (for example, by providing a taxonomy to distinguish between different categories of implementation theories, models and frameworks) [3], it is important that information about how and why to select and apply theoretical approaches is made accessible to frontline health professionals who strive to provide the best quality, evidence-based care to their patients.

Throughout this manuscript, we will caution the reader that this article cannot provide a simple “paint by numbers” approach to implementation. It would be counter-productive to try to create an algorithm that would capture planning and implementing behaviour change across multiple innovations in the complex adaptive systems of modern healthcare. Rather, we encourage thoughtful assessment and responsiveness to the particular circumstances of each implementation—the change to be introduced, the proposed way to make the change, the people who need to be involved, and the setting in which the change happens. Our intention is to provide accessible guidance based on our collective clinical and research experience, to assist clinicians and clinical researchers in their endeavours to design and conduct more effective implementation projects to improve clinical care, and to design more robust evaluations to advance the empirical evidence for this emerging science.

Therefore the aims of this paper are to:

- Demystify some of the jargon through defining the nature and role of frameworks, models and theories
- Describe and compare commonly used theoretical approaches, and how they are applied in practice
- Suggest how to select a theoretical approach based on purpose, scope and context of the individual project

The suggestions made in this debate paper are derived from our experiential knowledge as a multidisciplinary group of clinicians and health service researchers with interest and experience in implementation science in different international settings. EAL was a hospital-based physiotherapist for 14 years and is now an early-career researcher, AM is a practicing general physician and mid-career researcher, SK has a psychology background and is a researcher in a National Institute of Health Research Collaboration for Leadership in Applied Health Research and Care, SCH (psychology background) is an early career researcher, and GH and ALK have backgrounds in nursing and are senior researchers with extensive experience in improvement and implementation research. We work collaboratively with front-line clinicians and run workshops to help clinicians and clinical researchers apply theory to improve the success and sustainability of implementation projects. Through this work we recognise the need to assist clinicians and clinical researchers to navigate this complex landscape.

We encourage the reader to consider our recommendations as prompts or guides rather than definitive prescriptions. We have found that a more systematic understanding of implementation processes tends to be generated when different people (with different backgrounds and different approaches to implementation) share their experiences. Therefore, we have framed this paper using the experiences of author AM to illustrate the ways that clinicians can engage with utilising implementation theories, models and frameworks.

*Case study part 1 (experience of author AM):* I am a physician and I plan to implement a delirium prevention program. I have some implementation experience and know that it won’t be easy. I have heard about implementation science, so I hope there may be tools to help me.

I understand a bit about *Knowledge to Action* (KTA) to guide my planning. I have strong evidence of effectiveness and cost-effectiveness *[knowledge creation & synthesis]*, and there are established clinical practice guidelines *[knowledge tools/products]*. There is an effective model to implement delirium prevention developed in the USA (http://www.hospitalelderlifeprogram.org), but it used skilled geriatric nurses and large numbers of trained volunteers, which is not feasible in my hospital. None of the strategies in the guidelines are “hard” but they just don’t seem to get done consistently. I need to find out from staff and patients why this is the case, and then try to find ways to support them. Perhaps they need more education or reminders, or maybe we can reallocate the tasks to make it easier? Or are there strategies I am not familiar with? Whatever I do, I want to measure better care in some way to keep my boss happy and the staff interested. And my previous projects have tended to fizzle out over time… KTA gives me part of a plan but I need some more tools to know how to take the next steps.

## Main text

### Defining frameworks, models and theories

Some researchers have delineated between frameworks, models and theories, whereas other researchers use these terms interchangeably. In general, implementation frameworks, models and theories are cognitive tools that can assist a researcher or implementer to plan, predict, guide or evaluate the process of implementing evidence into practice.

Generally (for more detail refer to cited reference) [5]:

- A framework lists the basic structure and components underlying a system or concept. Examples of typical frameworks are the Consolidated Framework for Implementation Research (CFIR) [6], the Theoretical Domains Framework (TDF) [7, 8], RE-AIM [8–10] and Promoting Action on Research Implementation in Health Services (PARIHS) [9, 10].
- A model is a simplified representation of a system or concept with specified assumptions. An example of a model is the Knowledge to Action (KTA) cycle [11].
- A theory may be explanatory or predictive, and underpins hypotheses and assumptions about how implementation activities should occur. An example of a theory is the Normalization Process Theory (NPT) [12].

In our experience, clinicians and clinical researchers want to know what implementation approach will help them and their project best; for many clinicians and clinical researchers that we talk to, navigating the rapidly expanding number of implementation theories, frameworks and models is completely daunting, and is made worse by unfamiliarity with the language used and inconsistencies in nomenclature. To avoid compounding this problem, we will refer to frameworks, models and theories collectively as “theoretical approaches”, and support our readers to focus on which theoretical approach can best suit the purpose, scope and context of the implementation project.

Theoretical approaches help to shape how we think, which is why they are important. However, most of the time we are not aware of the underlying theories or frameworks we use. In implementation science, theoretical approaches have been developed for different purposes, with different intended users and are often underpinned by different philosophical perspectives (see Table 1). For instance, some have been designed to assist implementation researchers and enhance the quality of implementation research, [6] to support improvement and practice development in clinical settings [9] to understand factors influencing the implementation of evidence in particular health service settings [13] or to ensure comprehensive evaluation or reporting of an implementation program [14]. Some are based on the underlying assumption that implementation is rational and predictable when relevant factors are accounted for [7, 8]; in contrast, others are built on the assumption that implementation is unpredictable, and ongoing monitoring, flexibility and adaptation is required [9, 10]. Some have been designed iteratively, based on the developers’ experience in implementing evidence in real-world settings [9, 15, 16], whereas others have been developed systematically through reviewing and synthesising published literature [6, 7, 11]. And finally, some but not all theoretical approaches have been tested, validated and/or adapted over time [8, 10, 17].

**Table 1:** Summary of ten commonly applied theoretical approaches to implementation

Knowledge to Action [11]	
Purpose (as described by authors) A framework to conceptualise the process of knowledge translation which integrates the roles of knowledge creation and knowledge application. Provide conceptual clarity by offering a framework to elucidate the key elements of the knowledge translation process	
Brief description: This approach provides an overview to help guide and understand how knowledge is created and synthesised, and tools (like clinical guidelines) are developed, then how these tools are applied in clinical settings through tailoring and adaptation, implementation, monitoring and sustaining. Assumes that action plans will be realised (underpinned by assumption that actions are rational). Takes a systems approach – recognises that knowledge producers and users are situated within a larger social system	
How developed: Developed by reviewing literature of > 30 planned action theories, identified common elements. Added to planned action model a knowledge creation process and labelled the combined models the knowledge to action cycle.	
Changes/developments over time: No	
Ease of use: clear and easy to understand, intuitive. No specific guidance on how to do each step of the action cycle but provides some guidance on important elements to consider.	
Additional resources: no specific resources currently available on how to action each step of cycle	
Theoretical Domains Framework (TDF) [7, 8]	
Purpose (as described by authors): An integrative theoretical framework, developed for cross-disciplinary implementation and other behaviour change research to assess implementation and other behavioural problems and to inform intervention design.	
Brief description: provides a holistic list of factors that influence behaviour – application of TDF can give researcher confidence that factors influencing an individual’s behaviour will be identified, which in turn can identify factors that need to be addressed in order for behaviour change to occur (i.e. can be used to inform behaviour change strategy development/selection). Can be used in conjunction with Behaviour Change Wheel to develop and deliver behaviour change strategy	
How developed: through an expert consensus process and synthesis of 33 theories and 128 key theoretical constructs related to behaviour change.	
Changes/developments over time: Validity was investigated by behavioural experts sorting theoretical constructs using closed and open sort tasks. Validation study demonstrated good support for the basic structure of the TDF and led to refinements, leading to publication of new iteration of framework in 2012	
Ease of use: Quite straightforward to apply, can be time consuming to use for analysis – potential to overwhelm novice researcher given the 14 domains and 84 component constructs. COM-B and Behaviour Change Wheel work together with TDF.	
Additional resources: interview guides provided in publications [7, 30] assist ease of data collection and illustrate domains. Subject of thematic series in *Implementation Science* journal, guide to use of TDF published 2017 [31].	
RE-AIM framework [14, 17]	
Purpose (as described by authors): Originally developed as a framework to guide consistent reporting of evaluations regarding the public health impact of health promotion interventions, thereby providing a framework for determining what programs are worth sustained investment and for identifying those that work in real-world environments.	
Brief description: Reporting checklist for public health interventions (what patient groups are receiving intervention, have patient outcomes changed, what health professionals/ health professional groups are providing intervention, are they delivering intervention as intended, will the program be sustained in the long term) to evaluate real world impact. Can be used when designing or evaluating a public health intervention.	
How developed: Through inductive thinking building on results of previous research	
Changes/developments over time: “E” was initially efficacy [14], then effectiveness [17]	
Ease of use: Easy, interventions can be rated on the five dimensions, providing a score. Some of the reporting points (in particular Reach and Adoption) are not being interpreted and reported as developers intended	
Additional resources: dedicated website with online tools, examples [33]	
Consolidated Framework for Implementation Research [6]	
Purpose (as described by authors): Framework to promote implementation theory development and verification about what works, where and why.	
Brief description: list of factors (5 domains and 37 constructs) that can influence an implementation project, can be used in planning or in evaluation stages (does not guide how to implement). Research focus in contrast to doing/practitioner focus	
How developed: Published theories which sought to facilitate translation of research findings into practice in the healthcare sector were reviewed. Team identified constructs that had evidence that they influenced implementation and could be measured. Some constructs were streamlined and combined, whereas other constructs were separated and delineated.	
Changes/developments over time: No	
Ease of use: Clear, but may be difficult to digest language if new to area of implementation science	
Additional resources: dedicated website that provides examples, templates and tools to assist in developing and evaluating implementation projects, collecting and analysing data [28]	
Conceptual model of evidence-based practice implementation in public service sectors [15]	
Purpose (as described by authors): A multi-level, four phase model of the implementation process that can be used in public service sectors.	
Brief description: Conceptual model of factors that can influence implementation in the unique context of public sector services (focus on role of service delivery organisations and the services in which they operate) at each of the 4 implementation stages: Exploration, Adoption/Preparation, Implementation, Sustainment (EPIS). Explicitly recognises that different variables play crucial roles at different points in the implementation process. Does not provide guidance on how to move through different stages of implementation.	
How developed: based on literature and authors’ experience of public service sectors, funded by the National Institute of Mental Health	
Changes/developments over time: No	
Ease of use: Little clarity on how to operationalise different factors, potential to be confusing for those unfamiliar with implementation	
Additional resources: California Evidence-Based Clearinghouse for Child Welfare have developed webinars regarding use of EPIS framework. Freely available from http://www.cebc4cw.org/implementing-programs/tools/epis/	
Conceptual model of implementation research [19]	
Purpose (as described by authors) a heuristic skeleton model for the study of implementation processes in mental health services, identifying the implications for research and training.	
Brief description: Guides how implementation research can be organised, how it fits/aligns with evidence-based practices. May be useful for complete novice who needs clarity between clinical interventions, implementation strategies, and working through how to measure clinical and implementation effectiveness. Various theories can be placed upon the model to help explain aspects of the broader phenomena.	
How developed: drawn from 3 extant frameworks: stage pipeline model, multi-level models of change and models of health service use.	
Changes/developments over time: No	
Ease of use: Clear and easy to understand	
Additional resources: No	
Implementation effectiveness model [16]	
Purpose (as described by authors): an integrative model to capture and clarify the multidetermined, multilevel phenomenon of innovation implementation	
Brief description: A list of constructs that can influence implementation effectiveness, based on the premise that implementation effectiveness is a function of an organisation’s climate for implementing a given innovation and the targeted organisational members’ perceptions of the fit of the innovation to their values. Does not provide specific guidance for how to implement, was not designed specifically for the context of health care. Likely to be most useful for projects with a clear organisational approach.	
How developed: from authors’ personal experience with reference to literature	
Changes/developments over time: No	
Ease of use: Main manuscript very wordy (text-based). Concepts are clear.	
Additional resources: No	
Promoting Action on Research Implementation in Health Services (PARIHS) [9, 10]	
Purpose (as described by authors): Organisational or conceptual framework to help explain and predict successful implementation of evidence into practice and to understand the complexities involved.	
Brief description: Conceptualises how evidence can be successfully implemented in health care settings using the process of facilitation. Underlying premise is that facilitation will enable people to apply evidence in their local setting, which is situated within a broader organisational and societal context. Framework strives to capture the complexities involved in implementation, so most useful in more complex projects.	
How developed: from authors’ experience working as facilitators and researchers on quality improvement activities and health service research projects.	
Changes/developments over time: Has had several iterations since first publication in 1998 in response to findings from empirical testing. Revised to integrated or i-PARIHS framework in 2015 [10]	
Ease of use: Does not operationalise its constructs, so may be difficult for novice to understand and apply, particularly when not being supported by expert facilitator. Facilitator’s toolkit easy to apply to conduct pre- and post-implementation evaluation. For people experienced in implementation, framework provides guidance on all of the things to consider when implementation is complex.	
Additional resources: Facilitator’s Toolkit in book associated with 2015 iteration of PARIHS guides user through how to assess, facilitate and evaluate [32]	
Interactive Systems Framework [13]	
Purpose (as described by authors): Heuristic to help clarify the issues related to how to move what is known about prevention (particularly prevention of youth violence and child maltreatment) into more widespread use.	
Brief description: Framework regarding translating findings from prevention research to clinical practice. The framework comprises three systems: the Innovation Synthesis and Translation System (which distils information about innovations and translates it into user-friendly formats); the Innovation Support System (which provides training, technical assistance or other support to users in the field); and the Innovation Delivery System (which implements innovations in the world of practice).	
How developed: Collaborative development of the framework by Division of Violence Protection staff members, university faculty and graduate students, with input from practitioners, researchers, and funders.	
Changes/developments over time: No	
Ease of use: Easy to understand, no clear guidance available regarding how to apply framework	
Additional resources: No	
Normalization Process Model, Normalization Process Theory (NPT) [12]	
Purpose (as described by authors): provides a conceptual framework for understanding and evaluating the processes by which new health technologies and other complex interventions are routinely operationalized in everyday work, and sustained in practice.	
Brief description: NPT is an Action Theory, which means that it is concerned with explaining what people do rather than their attitudes or beliefs. Proposes that for successful sustained use: individuals & groups must work collectively to implement intervention; work of implementation occurs via 4 particular processes; continuous investment carrying forward in space and time required. Can be helpful to understand and evaluate how new health technologies/complex interventions are routinely operationalised sustained in practice. Not designed to guide implementation.	
How developed: in iterations, based on experiences of authors. Initially, developers mapped the elements of embedding processes and developed the concept of normalization. Next a robust applied theoretical model of Collective Action was produced, and applied to trials, government processes and healthcare systems. The final stage focused on building a middle-range theory that explains how material practices become routinely embedded in their social contexts.	
Changes/developments over time: Through its focus on being a theory, the authors continually refine and test NPT to ensure its validity. More recently, NPT has been extended towards a more general theory of implementation.	
Ease of use: easy to apply with use of specifically developed resources	
Additional resources: dedicated website with toolkit, examples. Interactive toolkit can be used to plan project or analyse data [29].	

### Commonly used theoretical approaches

Two articles were published in 2017 which presented the most commonly used dissemination and implementation research frameworks cited in academic publications [18] and the theories most commonly used by implementation scientists [2]. For pragmatic reasons (acknowledging the systematic approach taken by authors of both manuscripts), we used these two articles to guide the selection of theoretical approaches for discussion in this paper. We included the ten theoretical approaches that were within the top 15 on both lists (i.e. both highly cited in the literature and commonly used in implementation practice) [6–9, 11–16, 19]. These are presented in Table 1. We do not infer that these are the best or only theoretical approaches that should be used in implementation projects; simply that they are the most commonly used.

Of note, there are similarities across the theoretical approaches. All consider the ‘new evidence’ to be implemented; the ‘context’ where the evidence will be introduced; the ‘agents or actors’ who will use or apply the new evidence; and the ‘mechanisms’ or processes that actually make the changes happen. Mechanisms can either be people such as change champions, knowledge brokers, opinion leaders, project managers or facilitators or they can be processes such as new protocols, education sessions or audit and feedback cycles, or a combination of both.

It is important to acknowledge that there is no universally agreed-upon theory of successful implementation, nor empirical evidence about the relative advantages of one theoretical approach over another. While this may be frustrating to people new to the area of implementation science, the number of viable theoretical approaches offers clinicians an opportunity to “think outside the box”, and highlights the importance of clarifying what they are seeking to know or change through their project, and then being strategic in selecting a suitable theoretical approach.

*Case study part 2 (experience of author AM)*: So it is clear that I will need to adapt principles and protocols from successful programs in the USA to my local context. But how do I know what the context is? Top picks on Google scholar for “context assessment implementation science” seem to be *Consolidated Framework for Implementation Research* (CFIR) and *Promoting Action on Research Implementation in Health Services* (PARIHS). Both have nice guides that suggest useful questions to ask. There seems to be quite a lot of overlap, although I am drawn to PARIHS because from my experience I know that someone will need to spend time on the ward and build trust before we start to ask questions and introduce change. I suspect this ‘facilitator’ will be a critical role for the complex intervention because there are several behaviours to change.

When I look up “behaviour change implementation science”, the *Theoretical Domains Framework* (TDF) dominates. Like CFIR and PARIHS, there are a lot of elements, but I can see that they would be helpful for planning or analysing surveys and interviews with patients and staff to clarify what motivates, helps and hinders them. I do feel worried about how I will collect and analyse so much data across the several different groups involved in my project.

And once we have a thorough understanding of the context, staff and patients, how will I select strategies?

And if it does work, how long will it will take until the “new” becomes “normal” so that I can move on to the next problem? My colleague tells me that *Normalization Process Theory* (NPT) is a useful way to think about this, and I am impressed with the Normalisation of Complex Interventions-Measure Development (NoMAD) tool I find on their website; I can see how I could adapt it to find out whether staff feel the changes are embedded.

So where do I go from here? Do I frame the whole project with KTA, assess context with CFIR, assess the patient and staff views with TDF, adopt facilitation as the central element from PARIHS, and then look at how well it has gone using NPT? Am I being thorough or theoretically sloppy? They all look sensible, but I am not sure how to use any of them and I am worried it is going to add a whole lot of work to a complicated project.

### How the theoretical approaches have been used

Recent work investigating how theoretical approaches are selected suggests that theories are not selected to suit the purpose of the implementation research; rather theoretical approaches that are familiar to researchers tend to be used, regardless of the aim of the project [2]. To explore how theoretical approaches have been applied in the literature to date, we searched for and identified review papers for 6 of our 10 theoretical approaches: KTA [20], the Reach, Effectiveness, Adoption, Implementation, and Maintenance Framework (RE-AIM) [21], CFIR [22], NPT [23], PARIHS [24] and TDF [25]. (For details of these review papers, see Additional file 1: Table S1).

The overall message from these reviews is that theoretical approaches are not being utilised to their full potential. Despite the fact that many approaches have been developed to prospectively design and plan implementation strategies, they are almost overwhelmingly applied retrospectively to evaluate or interpret findings from a completed implementation project [22–24]. Further, the components of the theoretical approaches (such as coding systems or reporting dimensions) tend not to be applied consistently, with some users selecting to apply only particular theoretical components [21, 22], or applying components in different ways than the developers intended [21].

These findings again suggest that there is not an agreed “best” – or even “easiest” – theory to apply, and that even implementation researchers may need to take a pragmatic approach to the use of theory in complex real-world projects. It reflects the relative immaturity of the field, but this provides opportunities for clinical and research partners to contribute to advancing our knowledge of how theory is selected and adapted for practical use. To support thoughtful use of theory, we suggest some practical guidance to how to choose which theory to use, and to how to use the theory effectively to support the implementation project, based on our experience and interactions with clinical and academic staff new to implementation.

### How do you select the theoretical approach for your implementation project?

Research reporting guidelines for implementation studies, including Standards for Reporting Implementation Studies (STaRI) [26] and Template for Intervention Description and Replication (TIDieR) [27] specify that the rationale and theory underpinning implementation strategies should be reported. Some researchers also recommend that the reason for selecting a particular theoretical approach should be justified [2, 24].

We acknowledge that there will always be multiple questions that could be posed for each implementation project, each of which could be approached from different theoretical perspectives. Below we provide some prompts to assist clinicians and clinical researchers to select a theoretical approach that can value-add to different implementation projects, rather than simply citing a theoretical approach in order to meet a reporting guideline. Clinicians tend to be pragmatic; they generally are not motivated by concepts like theoretical purity, they just want things to work. We emphasise that there is an “art” to selecting and applying theoretical approaches – these prompts need to be applied and considered alongside a clinician or researcher’s experience and skill and the nuances of the implementation project.

**Fig. 1:**
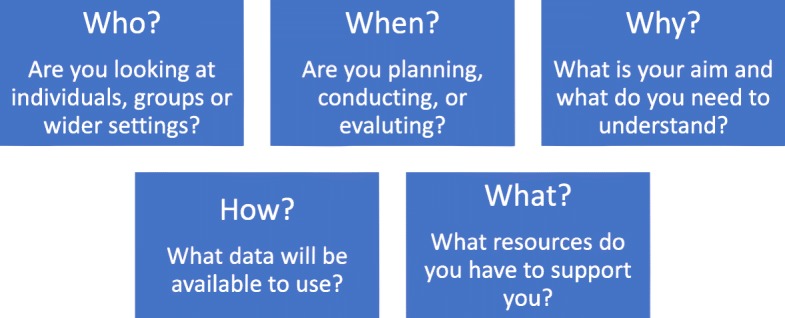
Five questions to help select a theoretical approach

In our experience, clinicians are often anxious that they will select the ‘wrong’ theory. We reiterate that there is no precise formula for choosing a theoretical approach. One important thing to consider in theory selection is the goodness-of-fit, which is determined by each study’s needs and aims, rather than there being a ‘wrong’ choice. The following are suggested questions that could be considered to identify which theoretical approach is particularly appropriate or useful for different implementation projects (see Fig. 1).

#### Who are you working with?

Are you working with individuals who have complete autonomy, are you working with a team, or are you working with an entire health service? Almost all implementation studies will inevitably touch on different organisational levels (micro-, meso- and macro-level implementation), so consider the fit of the theoretical approaches to the organisational level where your project is positioned, and whether more than one approach is required to guide implementation at different levels. Some approaches are particularly concerned with individual experiences or behaviours (for example, TDF), others with group interaction or collective working (for example, NPT; Klein) and others encompass the broader contextual factors impacting across a wider setting or service (for example, PARIHS).

#### When in the process are you going to use theory?

The point in time of the implementation project may be another factor guiding theoretical approach selection. Some approaches lend themselves particularly to the design and planning of an implementation strategy (for example, Exploration, Preparation, Implementation and Sustainment (EPIS); Proctor; Interconnected Systems Framework (ISF); TDF in conjunction with Behaviour Change Wheel), others to tracking the development of a project (for example, KTA), and others to planning an evaluation and defining outcome measures to assess implementation success (for example, RE-AIM).

#### Why are you applying a theory?

The aims and intended outcomes of each study should be considered, as different theoretical approaches provide different ‘pay offs’ in terms of the understanding gained. Different theoretical approaches can be used to measure achievement of a specific change (for example, TDF; ISF), to generate a better understanding of barriers and facilitators to inform implementation approaches (for example, PARIHS; CFIR), to develop knowledge about an ongoing implementation process (for example, KTA; Proctor), or to provide a framework of relevant implementation outcomes (for example, CFIR; RE-AIM).

#### How will you collect data?

Choice of theoretical approach may also be informed by what data will be available for analysis. Although ideally data collection would be designed with a particular approach in mind so that data are collected to answer the questions of interest, we are aware that in practice clinicians need to work with the resources that are available to them. For example, clinicians may have access to routinely collected outcome data which could be evaluated using the constructs in RE-AIM, but these same data might provide limited insight into the underlying mechanisms of action that NPT explores. Similarly, many services routinely collect qualitative data about professional or patient experiences which could be explored using an approach such as NPT, but these data would be unlikely to achieve a satisfactorily robust evaluation using the questions posed by RE-AIM. Again, we reiterate that this paper is not written as a prescriptive piece (i.e. we are not advocating that RE-AIM should be used to guide analysis of all projects with outcome data and NPT used to analyse projects with available patient and professional experience data) but these examples are given to illustrate the importance of careful selection of theoretical approach.

#### What resources are available?

The experience of the people who will be involved in coordinating the implementation project should be considered. People who have less experience in implementation projects might require structured tools to collect and analyse data, such as those developed for use with some approaches (for example, CFIR [28]; NPT [29]; TDF [30, 31], PARIHS [32] RE-AIM [33]). The number of staff, and the time available to them to participate in the implementation project should be considered – for example facilitation (a core component of PARIHS) requires a substantial time investment for one or more person to act as facilitator, whereas approaches that are more aligned to strategies such as training and audit and feedback sessions (for instance, EPIS) might be easier to implement with less staff or resource support.

#### Other questions to ask to help in choosing

- Does the theoretical approach have particular ‘face validity’ for the implementation project? For example, people interested in facilitation may recognise PARIHS as particularly relevant, or a project aiming to address motivations for behaviour change may lend itself to TDF.
- Does the theoretical approach draw your attention to aspects of implementation that you may have otherwise neglected? For example, implementing a large scale public health intervention may have varied success due to challenges meeting the most vulnerable populations, or providing the intervention as intended across a range of sites—features that can be captured effectively by RE-AIM.
- What theoretical approach(es) have studies in your topic area used? This can be helpful in providing worked examples of how particular theoretical approaches have been applied to add understanding to a project.

### How can theoretical approaches be used during your implementation project?

**Fig. 2:**
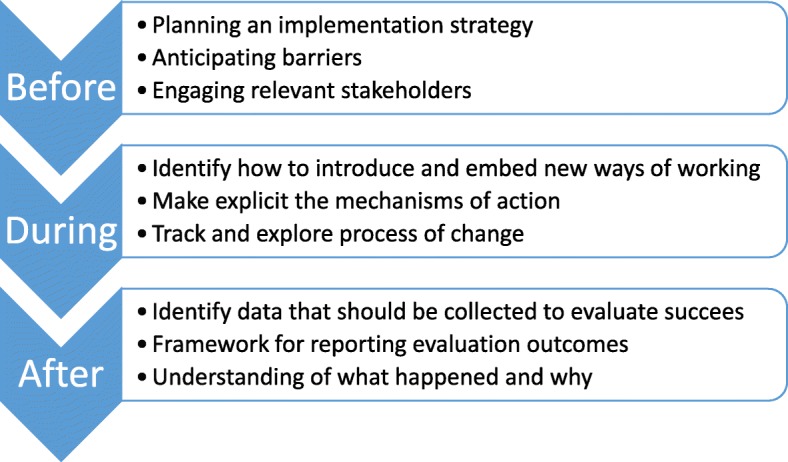
How a theoretical approach can support a project at different stages

Given that implementation science is designed to have the end-point of improved health processes and outcomes, clinicians and clinical researchers should be pro-active in applying theoretical approaches to guide planning, doing and evaluating implementation projects (see Fig. 2).

When developing an implementation plan, theory can be used to guide identification of key stakeholders (for example who should ideally participate in the implementation project; who needs to know about the program to ensure organisational support) and to guide the collection of pre-implementation data (for example details about the target practice or evidence to be implemented; details about the key stakeholders; features of the context in which implementation is to occur). Through identifying key stakeholders and collecting pre-implementation information, theoretical approaches can then guide the development of project-specific implementation strategies.

As well as guiding these practical steps of an implementation program, theoretical approaches can be used to predict or explore the process of change, through the development of logic models or program theories. In this way, the intended mechanisms of action can be made explicit, which can be useful when communicating with the key stakeholders, as well as exposing any hidden assumptions which may influence the success of the project.

Theoretical approaches can be used during an implementation program to guide how to introduce, embed and sustain new practices, with varying levels of guidance offered by the different approaches.

And lastly, theoretical approaches can be used to evaluate the success (or otherwise) of an implementation program through guiding what data should be collected, how results should be reported, and providing structure to guide analysis. Depending on the theoretical approach chosen, analysis may focus on the evidence being implemented, the people involved, the context in which implementation occurred, the process of implementation itself or interactions between these different factors.

*Case study part 3 (experience of author AM):* I choose PARIHS because I need to work with a whole range of staff, with different views and roles within this complex intervention, and I can see the need for facilitation. I am mostly interested in something to guide the “doing” and as my colleague and I begin to work with the first ward, we find using a lens of implementation science helps us to understand more about what is and isn’t working, so that instead of getting frustrated we can reflect more objectively and search for flexible solutions. On the first ward we also use the framework for reflection and for guiding a simple evaluation [34], and this provides useful structure for planning and adapting our approach on the next ward where the people and context differ in important ways. This then gives us confidence to start training other facilitators to “read” the local teams and context as we conduct a large funded trial across several hospitals [35], providing evidence of transferability that will be critical for spread to improve outcomes at scale. We are able to capture more consistent data about the different wards we are working on, which help us understand inconsistencies in our results, and identify the most important factors that predict which wards can implement this program successfully to help us target scarce resources. We are also able to collect data about the facilitation process, and how facilitators learn this role. We find as we become more familiar with PARIHS it becomes more useful for planning, doing and evaluating our improvements.

But we are also involved in projects that this framework is too complicated to use in. Our experience makes us more confident to look for and try out other theoretical approaches that we think will suit those projects better, and help us achieve – and importantly understand – our outcomes.

## Conclusions

We have written this paper based on our collective clinical and research experience to assist clinicians and clinical researchers to plan, conduct and evaluate implementation projects. Ours is a pragmatic guide based on our experiences; we look forward to others’ work in the field who are using empirical research methods to investigate how to select and apply theoretical approaches. We anticipate there will be ongoing developments and refinements in implementation theory, so it is important to recognise the great opportunities that currently exist for the implementation research and clinical communities to come together to more explicitly co-design interventions based on sets of theoretical assumptions and predictions that can inform implementation projects. Imagine conversations around theory selection that connect into clinicians’ sense of their context and the challenges they face, their history and resources, and their understanding of the cultural barriers and drivers for change. These are all important factors that need to be integrated into any ‘proposed theory of implementation’ generated by the clinicians and their research partners.

We planned this paper to help clinicians and clinical researchers to become more familiar with the different implementation theoretical approaches, to understand their rationale and existing usage, and to have the confidence to apply theory to implementation projects. We encourage clinicians and novice researchers to be open minded and at the same time to trust their instincts when selecting a theoretical approach when conducting implementation projects, all the while being conscious to consider how the different approaches fit the aims, scope and resources available. In this way, the most useful approach will be applied, rather than using a familiar or previously-applied approach that does not value-add to the project at hand. We urge clinicians to consider and select the theoretical approach(es) prospectively to be of most benefit to the project, and we encourage anyone and everyone involved in implementation projects to reflect and share their experiences about whether (and how) the theoretical approach contributed to the conduct and success of the project. In doing so, clinicians, clinical researchers and implementation scientists can collectively close the gap between espoused theory and theory in use, which can help our community of health professionals and researchers to refine our thinking. This then will contribute to more nuanced advice on how to address evidence-practice gaps and ultimately to contribute to better health outcomes.

## Additional file

Additional file 1: **Table S1.** Review papers about use of theoretical approaches. This table provides summaries of published manuscripts which review use of 6 theoretical approaches. (DOCX 15 kb)

## Abbreviations

CFIR: Consolidated framework for implementation research
EPIS: Exploration, preparation, implementation and sustainment
i-PARIHS: Integrated promoting action on research implementation in Health Services
ISF: Interconnected systems framework
KTA: Knowledge to action
NHMRC: National health and medical research council
NoMAD: Normalisation of complex interventions-measure development
NPT: Normalization process theory
PARIHS: Promoting action on research implementation in health services
RE-AIM: Reach, effectiveness, adoption, implementation, and maintenance
STaRI: Standards for reporting implementation studies
TDF: Theoretical domains framework
TIDiER: Template for intervention description and replication
TRIP: Translating research into practice
