# Supplementary material for: “There is nothing so practical as a good theory”: a pragmatic guide for selecting theoretical approaches for implementation projects
Source: BMC Health Serv Res. 2018 Nov 14;18:857. doi: 10.1186/s12913-018-3671-z (PMC6236961; doi:10.1186/s12913-018-3671-z)
Supplement: Supplementary file 1 — Table S1. Review papers about use of theoretical approaches. This table provides summaries of published manuscripts which review use of 6 theoretical approaches. (DOCX 15 kb) [file 12913_2018_3671_MOESM1_ESM.docx]

Table S1

| **Type of review paper** | **Number of papers that used framework** | **Number of papers that meaningfully used** | **How the framework was used** | **How the framework was not used** |
| --- | --- | --- | --- | --- |
| Knowledge to Action  Field B, Booth A, Ilott I, Gerrish K: Using the Knowledge to Action Framework in practice: a citation analysis and systematic review. Implement Sci. 2014; 9:172 | | | | |
| Citation analysis and systematic review | 164 | 10 | Used to inform through to fully integrated into the project. | Not used to its full capacity – no one study used every phase of the framework. |
| Theoretical Domains Framework  Francis JJ, O'Connor D, Curran J. Theories of behaviour change synthesised into a set of theoretical groupings: introducing a thematic series on the theoretical domains framework. Implement Sci. 2012;7:35. | | | | |
| Thematical grouping/  tabling of studies | 133 | 21 | Considerable breadth - exploratory studies to identify barriers, questionnaire and interview studies, randomised trials, etc. | This framework’s focus on behaviour change theory means many poorly used or applied the framework without appropriate understanding or training. |
| Reach, Effectiveness, Adoption, Implementation, and Maintenance (RE-AIM) framework  Gaglio B, Shoup JA, Glasgow RE. The RE-AIM framework: a systematic review of use over time. Am J Public Health. 2013;103:e38-46. | | | | |
| Systematic review | 178 | 71 | Effectively used as a checklist to guide reporting of public health interventions. | The criteria and dimensions were not interpreted or reported in the way the authors intended – not all studies reported on all 5 dimensions and none of the studies utilised all 34 of the criteria. |
| Consolidated Framework for Implementation Research (CFIR)  Kirk MA, Kelley C, Yankey N, Birken SA, Abadie B, Damschroder L. A systematic review of the use of the Consolidated Framework for Implementation Research. Implement Sci. 2016;11:72. | | | | |
| Systematic review | 429 | 26 | The use of CFIR maps well onto its intended and suggested use (planning, guiding and evaluation).  It was also used to gain understanding of practitioners’ experiences of implementation (i.e. processes, barriers and facilitators). | Some studies utilised the domains and others utilised the domains and the constructs. Some studies incorrectly applied CFIR post-implementation, where their application was more aligned with the CFIRs pre-implementation guidance. |
| Promoting Action on Research Implementation in Health Services (PARIHS)  Helfrich CD, Damschroder LJ, Hagedorn HJ, Daggett GS, Sahay A, Ritchie M et al. A critical synthesis of literature on the promoting action on research implementation in health services (PARIHS) framework. Implement Sci. 2010;5:82. | | | | |
| Critical synthesis | 33 | 24 | Framework was utilised as a way to organise analyses. | None of the studies utilised the framework to prospectively design their implementation strategies, despite the frameworks utility to be used in this way. |
| Normalization Process Theory (NPT)  McEvoy R, Ballini L, Maltoni S, O’Donnell CA, Mair FS, MacFarlane A. A qualitative systematic review of studies using the normalization process theory to research implementation processes. Implement Sci. 2014;9:2-2. | | | | |
| Systematic review | 383 | 29 | Used to study the implementation processes of complex interventions and to develop tools to support the implementation process. | Some issues surrounding coding decisions as well that few studies reported their rationale for using NPT.  Some studies took a single-stakeholder perspective despite NPT endorsing a whole-systems approach.  Further, only one study used NPT prospectively – more should use it in this way. |
